# Advances in Plasma Oncology toward Clinical Translation

**DOI:** 10.3390/cancers12113283

**Published:** 2020-11-06

**Authors:** Abraham Lin, Katharina Stapelmann, Annemie Bogaerts

**Affiliations:** 1 Plasma Lab for Applications in Sustainability and Medicine–Antwerp (PLASMANT), Department of Chemistry, University of Antwerp, 2610 Antwerp, Belgium; 2 Center for Oncological Research (CORE), Integrated Personalized & Precision Oncology Network (IPPON), University of Antwerp, 2610 Antwerp, Belgium; 3 Department of Nuclear Engineering, North Carolina State University, Raleigh, NC 27695, USA

This Special Issue on “Advances in Plasma Oncology Toward Clinical Translation” aims to bring together cutting-edge research papers within the field in the context of clinical translation and application. Plasma oncology is a broad, multidisciplinary field encompassing plasma physics, redox chemistry, cancer biology and immunology, and various disciplines of engineering. Together, researchers in these different fields have worked together to investigate the interaction of cold atmospheric plasma (CAP) with cells and tissue and to develop CAP systems for clinical cancer treatment.

In the past decade, research in plasma oncology has been fruitful. CAP has been shown to kill a multitude of cancer cell lines in vitro using several different devices and delivery methods [1,2,3]. Thorough physical and chemical analysis of CAP, both in the air and liquid interfaces, have linked the anti-cancer effects of CAP to reactive oxygen and the nitrogen species it produces [3,4]. On the biological side, investigations into the interaction of these species with cells and tissue have also elucidated several activated intracellular pathways following exposure to CAP [5,6]. The efficacy of CAP treatment in living organisms is also becoming more evident, as more animal studies are being performed and its ability to reduce tumor burden and prolong animal survival is being demonstrated [7]. In these more complex models, the ability of CAP to affect anti-cancer immune responses is also emerging, which could have a profound impact on cancer immunotherapy and combination strategies [7,8,9]. Finally, the beneficial effects of CAP for oncology have also been reported in the first pilot clinical studies [10,11,12]. Altogether, it is clear that the past decade has brought significant progress to the field of plasma oncology through the collaborative and multidisciplinary efforts of researchers in various fields.

It is our hope that in the upcoming decade, the plasma oncology community will continue to progress by focusing on bench-to-bedside strategies. This will require focused investigation into fundamental research (e.g., variation in cancer types, tumor heterogeneity), utilization of advanced techniques linking the generation and transport of reactive species, and addressing of challenges and limitations in the clinic (e.g., standardization of treatment, strategic combination therapies). As the plasma oncology field continues to grow, we hope that the articles in this Special Issue will begin to cover these items, as well as bring to light other elements to be considered. Only by acting together in this global effort will CAP technology reach a standard of clinical safely and efficaciousness for the benefit of cancer patients.
